# 人肺腺癌Anip973/NVB耐药细胞系动物模型的建立及其耐药机制的研究

**DOI:** 10.3779/j.issn.1009-3419.2012.03.03

**Published:** 2012-03-20

**Authors:** 萌 王, 璇 洪, 钦文 孙, 瑞林 李, 朝阳 杨, 公琰 陈

**Affiliations:** 1 150081 哈尔滨，哈尔滨医科大学附属第三医院内一科 Department of Medical Oncology, the Third Affiliated Hospital of Harbin Medical University, Harbin 150081, China; 2 150001 哈尔滨，哈尔滨市胸科医院肿瘤科 Department of Medical Oncology, Thoracic Hospital of Harbin City, Harbin 150001, China; 3 264200 威海，威海市市立医院肿瘤科 Department of Medical Oncology, Hospital of Weihai City, Weihai 264200, China

**Keywords:** 肺肿瘤, 多药耐药, 去甲长春花碱, Bcl-2, MRP3, Lung neoplasms, Multidrug resistance, Navelbine, Bcl-2, MRP3

## Abstract

**背景与目的:**

肿瘤的多药耐药性（multidrug resistance, MDR）是导致肺癌化疗失败的主要原因，长春瑞滨（Vinorelbine，诺维本，NVB）是治疗非小细胞肺癌最有效的化疗药物之一，本研究旨在建立人肺腺癌Anip973/NVB耐药细胞的动物模型，并初步探讨其耐药机制。

**方法:**

采用皮下注射法建立人肺腺癌Anip973细胞和耐药细胞Anip973/NVB的裸鼠移植瘤模型，分成Anip973治疗组、Anip973对照组、Anip973/NVB治疗组、Anip973/NVB对照组。观察肿瘤生长情况，绘制生长曲线，计算抑瘤率；通过电镜观察移植瘤的细胞形态学变化；通过免疫组化法检测移植瘤中Bcl-2蛋白和MRP3蛋白的表达来进一步研究人肺腺癌Anip973/NVB细胞的耐药机制。

**结果:**

与空白对照组比较，Anip973细胞和Anip973/NVB细胞移植瘤经NVB治疗后抑瘤率分别为60.00%、4.65%，Anip973/NVB细胞移植瘤生长受抑无统计学差异；电镜显示：经NVB治疗后，Anip973细胞出现特异性的凋亡形态学特征改变，而Anip973/NVB细胞仍然呈肿瘤细胞生长的典型形态。免疫组织化学染色结果显示：Bcl-2和MRP3蛋白在Anip973/NVB细胞移植瘤中的阳性表达率均明显高于在Anip973细胞移植瘤中的表达（*P* < 0.001）。

**结论:**

Bcl-2及MRP3蛋白在Anip973/NVB耐药细胞裸鼠移植瘤内的高表达可能是该细胞系耐药的重要机制之一。

## Abstract (en)

**Background and objective:**

Multidrug resistance (MDR) is the main cause of chemotherapeutic failure in lung cancer, and vinorelbine (NVB) is one of the most efficient drugs that threaten non-small cell lung cancer (NSCLC). The current study aims to establish tumor xenografts and investigate the molecular mechanisms involved in the resistance of NVB in lung adenocarcinoma.

**Methods:**

Nude mice were implanted with Anip973 and Anip973/NVB, and tumor-bearing mice were divided into the Anip973 treatment, Anip973 control, Anip973/NVB treatment, and Anip973/NVB control groups, respectively. The current study observes tumor growth, draws growth curves, and calculates inhibitory rates. The morphological changes in cell tumor were observed through the immunohistochemical method using an electron microscope to detect the expressions of MRP3 and Bcl-2 and to investigate the molecular mechanisms of Anip973/NVB cells.

**Results:**

The tumor inhibitory rates of the Anip973 and Anip973/NVB cells treated with NVB were 60.00% and 4.65%, respectively, compared with the control group. The growth inhibition in the Anip973/NVB cell transplantation tumor had no significant difference. Apoptosis was observed using TEM when the Anip973 transplantation tumor was treated with NVB. On the other hand, no apoptosis was found in the Anip973/NVB transplantation tumor using TEM. Immunohistochemical staining (SP) shows the positive expressions of Bcl-2 and MRP3 proteins in Anip973/NVB transplantation tumor, which were observed to be higher than those in the Anip973 transplantation tumor.

**Conclusion:**

The overexpression of Bcl-2 and MRP3 might be one of the major mechanisms of the MDR of Anip973/NVB.

肺癌是严重威胁人类健康与生命的恶性肿瘤，其发病率和死亡率在许多国家均居恶性肿瘤之首。目前，化疗仍是晚期非小细胞肺癌（non-small cell lung cancer, NSCLC）的主要治疗手段，长春瑞滨（Vinorelbine，诺维本，NVB）是治疗NSCLC最有效的药物之一，而多药耐药（multidrug resistance, MDR）现象是其化疗失败的重要因素之一。迄今为止，在肺腺癌中关于NVB诱导的耐药机制尚未见过报道，本研究的前期研究结果^[1-3]^表明，Bcl-2和MRP3在Anip973/NVB细胞系中的表达上调，且Bcl-2在肝癌、骨肉瘤，小细胞肺癌的耐药机制中发挥了重要的作用。MRP家族中与肺癌耐药有关的主要是MRP1、MRP3，在NSCLC细胞系中MRP3的表达水平与肿瘤细胞对ADR、VCR、VP-16及DDP等化疗药物的耐药呈正相关^[4]^。

基于以上研究，本研究应用前期建立的Anip973/NVB的耐药细胞系（已申请专利），建立裸鼠移植瘤模型，通过检测Bcl-2和MRP3蛋白的表达来进一步研究人肺腺癌Anip973/NVB细胞系的耐药机制。

## 1 材料与方法

### 1.1 药物与试剂

NVB（法国皮尔法伯药物研制公司）；优级胎牛血清（中国医学科学院生物工程研究所）；RPMI-1640培养液（黑龙江省肿瘤研究所自行配制）；胰酶（黑龙江省肿瘤研究所自行配制）；兔抗人Bcl-2（N-19）多克隆抗体（美国Santa Cruz Biotechnology公司），工作浓度为1:200；兔抗人MRP3单克隆抗体（北京博奥森生物技术有限公司），工作浓度为1:300；PV-6001（北京中杉金桥生物技术有限公司）。

### 1.2 细胞株与动物

人肺腺癌细胞株Anip973由哈尔滨医科大学附属肿瘤研究所惠赠。人肺腺癌耐药细胞株Anip973/NVB由作者前期诱导建立^[5]^。

裸鼠（品系nu/nu），雌性，17 g-21 g，4周-6周龄，30只，北京动物研究所繁育，SPF级实验动物，实验动物合格证号SCXK（京）2007-0001。所用垫料、饮水、标准饲料及其它与动物接触的物品均经高压灭菌处理。

### 1.3 实验方法

① 皮下移植瘤模型的建立：采用培养细胞皮下接种移植法，用1 mL注射器将人肺腺癌细胞Anip973和耐药细胞Anip973/NVB以1×10^7^个/只接种1次至裸鼠左腋侧皮下，待瘤体长径长至约10 mm时开始实验；②动物：人肺腺癌细胞Anip973成瘤的裸鼠取12只完全随机化分为Anip973治疗组、Anip973对照组；人肺腺癌耐药细胞Anip973/NVB成瘤的裸鼠取12只完全随机化分为Anip973/NVB治疗组、Anip973/NVB对照组。每组6只，治疗组NVB注射剂量按20 mg/kg、0.2 mL生理盐水稀释，尾静脉注射，对照组注射0.2 mL生理盐水。治疗7 d后处死裸鼠，取下皮下肿瘤，称瘤质量。

### 1.4 观察指标

① 观察治疗前后瘤体的大小：以体积（mm^3^）=0.5×长径（mm）×短径（mm）^2^计算肿瘤体积，每天测量1次，绘制生长曲线；②计算抑瘤率（%）=（1-实验组平均肿瘤重量/对照组平均肿瘤重量）×100%，局部实体瘤生长体积的测定：注射前用游标卡尺测量肿瘤的最长径（a）和最短径（b），并称体重。肿瘤体积（V）=1/6пab^2^（п表示圆周率）；③形态学观察：取1 mm×1 mm×1 mm的新鲜肿瘤组织，用2%戊二醛固定，环氧树脂包埋，超薄切片机制片，醋酸铀和柠檬酸铅双重染色，透射电镜观察；④Bcl-2和MRP3蛋白的表达：方法：标本经10%福尔马林固定，常规脱水，石蜡包埋，切片作免疫组织化学染色（SP法）；一抗分别为：Bcl-2、MRP3；结果判断标准：Bcl-2胞浆着色为阳性，MRP3胞浆和（或）胞膜着色为阳性，按阳性细胞占肿瘤的比例，将 < 25%为阴性，≥25%且 < 50%为阳性，≥50%为强阳性。

### 1.5 统计学分析

应用SAS 9.1统计软件包进行统计分析，所有数据以Mean±SD表示，组间比较用析因设计的方差分析法，两两比较结果用*Bonferroni*法，两者相关性用*Pearson*相关系数（*r*）分析，以*P* < 0.05为有统计学差异。

## 2 结果

### 2.1 裸鼠生长及成瘤情况

整个实验过程中裸鼠饮水、进食正常，无腹泻、活动迟缓等不良反应，无一荷瘤裸鼠自然死亡（图 1）。Anip973细胞移植瘤成瘤率为93.3%（14/15），Anip973/NVB细胞移植瘤成瘤率为86.7%（13/15）。裸鼠在接种后平均6 d成瘤，接种10 d后肿瘤直径约10 mm。

**1:**
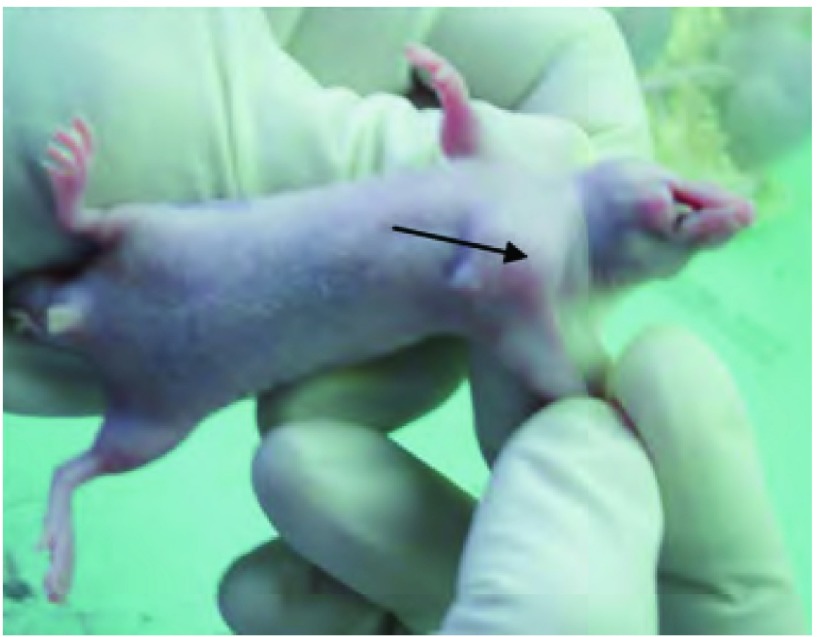
治疗中的裸鼠。箭头示瘤体。 Nude mice in the treatment. Arrow shows the tumor.

### 2.2 移植瘤的生长情况的比较

经NVB治疗后，Anip973治疗组移植瘤生长减缓甚至出现负增长，Anip973对照组、Anip973/NVB治疗组、Anip973/NVB对照组移植瘤生长曲线相同（图 2）；Anip973治疗组经NVB治疗的抑瘤率为60.0%，与Anip973对照组比较肿瘤生长明显受抑（*P* < 0.001）。Anip973/NVB治疗组的抑瘤率为4.65%，肿瘤生长与Anip973/NVB对照组比较无明显差异（*P*=0.358）（表 1）。

**2:**
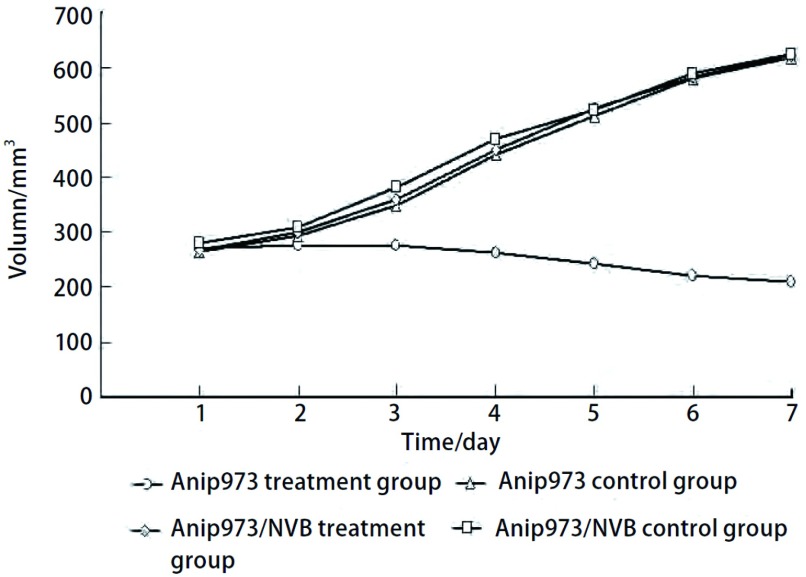
Anip973和Anip973/NVB细胞裸鼠移植瘤生长曲线。NVB治疗后，Anip973治疗组细胞移植瘤生长缓慢并且抑制了移植瘤的生长（*P* < 0.001）。然而，Anip973/NVB移植瘤经NVB治疗后，生长情况与两个对照组相似（*P*=0.358）。 Tumor growth curves of Anip973 cell and Anip973/NVB cell xenografts. After treated by vinorelbine, Anip973 xenografts grew slowly and vinorelbine inhibited the growth of Anip973 xenografts (*P* < 0.001). However, growth of Anip973/NVB xenografts treated by vinorelbine was similar with the two untreated groups (*P*=0.358).

**1:** 各组瘤重及抑瘤率的比较 Comparison of tumor weight and inhibition rate among groups

Group	*n*	Tumor weight (g)	Inhibition rate (%)
Anip973 treatment group	6	3.6±0.7	60.0
Anip973 control group	6	9.0±1.0	-
Anip973/NVB treatment group	6	8.2±1.0	4.65
Anip973/NVB control group	6	8.6±1.2	-

### 2.3 细胞形态学观察

透射电镜下Anip973细胞移植瘤经NVB治疗后细胞呈现凋亡的形态。细胞体积缩小，微绒毛结构消失，胞膜完整，可见部分细胞核仁消失。部分细胞胞质内还可见高密度团块物质以及凝聚细胞器的堆积（图 3A）。高倍下细胞胞质内充满大量的游离核蛋白体，线粒体少，粗面内质网呈小杆状或囊泡状，还可见大的胞内微腺腔结构，在细胞周围还可见吞噬细胞的碎片，已形成由质膜包裹内含完整的细胞器和核碎片等细胞内容物的凋亡小体（图 3B）。Anip973/NVB细胞移植瘤经NVB治疗后细胞形态不规则，表面有大量的微绒毛结构，核仁明显较多，胞质内有丰富的细胞器结构（图 3C）。高尔基复合体发达，线粒体较小，还可见到微腺管结构（图 3D）。

**3:**
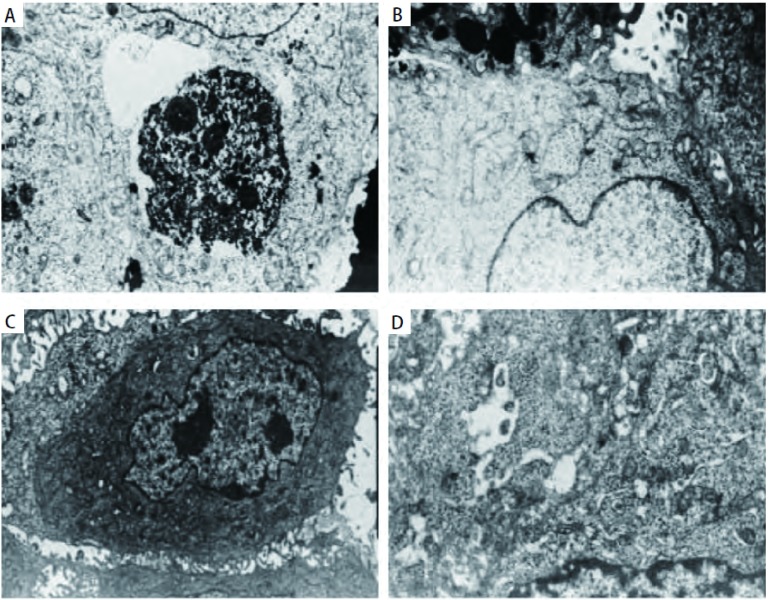
NVB治疗后Anip973和Anip973/NVB细胞透射电镜图（×10, 000）。A：透射电镜下Anip973移植瘤细胞经NVB治疗后细胞呈现凋亡的形态。细胞体积缩小，微绒毛结构消失，可见部分细胞核仁消失；B：Anip973移植瘤细胞经NVB治疗后染色质凝聚并形成凋亡小体；C：Anip973/NVB细胞移植瘤经NVB治疗后，表面有大量的微绒毛结构，核仁明显较多，胞质内有丰富的细胞器结构；D：Anip973/NVB细胞移植瘤经NVB治疗后高尔基复合体发达，线粒体较小，还可见到微腺管结构。 Morphological changes in apoptosis observed in Anip973 and Anip973/NVB xenografts after treatment with vinorelbine by TEM (×10, 000). A: Anip973 cells after treatment with vinorelbine presented apoptotic characteristics, such as cell shrinkage, loss of microvilli and nucleolus; B: Anip973 cells after treatment with vinorelbine presented chromatin condensation and formation of apoptotic bodies; C: Anip973/NVB xenografts after treatment with vinorelbine, the surface has a lot of microvilli structure, nucleuos increased clearly, there were rich cytoplasmic in the cytoplasm; D: Anip973/NVB xenografts after treatment with vinorelbine, presented developed golgi complexes, mitochondria was small, and micro-tublar structure can be seen.

### 2.4 Bcl-2蛋白和MRP3蛋白的表达

Bcl-2在Anip973/NVB治疗组、Anip973/NVB对照组移植瘤中的阳性表达率分别为74%、72%，明显高于在Anip973治疗组、Anip973对照组中的47%、48%（*P* < 0.001）。MRP3在Anip973/NVB治疗组、Anip973/NVB对照组移植瘤中的阳性表达率分别为79%、76%，也明显高于在Anip973治疗组、Anip973对照组移植瘤中的53%、52%（*P* < 0.001）（表 2）。Bcl-2和MRP3的表达无相关性（*r*=0.106, *P*=0.091）（图 4）。

**2:** Bcl-2和MRP3蛋白在Anip973和Anip973/NVB细胞裸鼠移植瘤中的表达 The expression of Bcl-2 and MRP3 in Anip973 and Anip973/NVB xenografts

Group	*n*	Expression of Bcl-2 (%)	Expression of MRP3 (%)
Anip973 treatment group	6	47.2±1.0	53.1±1.2
Anip973 control group	6	48.6±1.4	52.5±1.6
Anip973/NVB treatment group	6	74.3±1.5	79.6±1.1
Anip973/NVB control group	6	72.4±1.3	76.2±2.6

**4:**
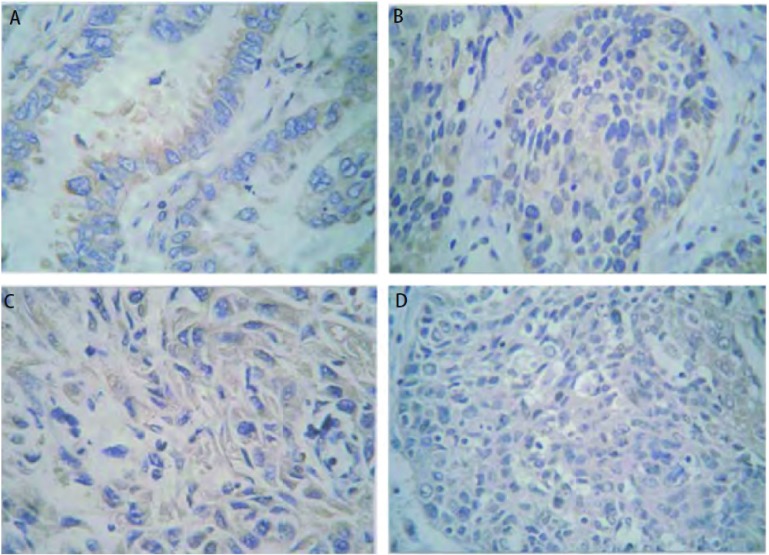
免疫组化法检测Bcl-2（A，B）和MRP3（C，D）蛋白在Anip973和Anip973/NVB细胞裸鼠移植瘤中的表达（x400）。Bcl-2蛋白的表达在Anip973/NVB治疗组（B）高于Anip973治疗组（A）。MRP3蛋白的表达在Anip973/NVB治疗组（D）高于Anip973治疗组（C）。 The expressions of Bcl-2 (A, B) and MRP3 (C, D) in Anip973 and Anip973/NVB xenografts by immunohistochemistry (x400). The expression of Bcl-2 in Anip973/NVB treatment group (B) was higher than that in the Anip973 treatment group (A), and the expression of MRP3 in the Anip973/NVB treatment group (D) was higher than that in the Anip973 treatment group (C).

## 3 讨论

肿瘤细胞对抗肿瘤药物产生耐药是导致肿瘤化学治疗失败的重要原因之一，也是困扰肿瘤治疗的关键性难题。NVB作为重要的抗癌药物，广泛应用于NSCLC、晚期乳腺癌、霍奇金病、小细胞肺癌等。研究^[5]^表明，多数细胞毒制剂通过诱导凋亡来杀伤细胞治疗肿瘤。Bcl-2家族是目前最受重视的调控细胞凋亡的基因家族，肺癌细胞耐药与凋亡途径中Bcl-2过度表达相关^[6]^。此外，Bcl-2在许多肿瘤中与化疗药物耐药密切相关^[7]^。在急性髓细胞样白血病中，Bcl-2与化疗药物耐药相关，并且针对Bcl-2 mRNA的反义寡核苷酸增加了AML细胞对表阿霉素的敏感性^[8]^。在胰腺癌细胞中Bcl-2过表达并通过ERK/Bcl-2通路抵抗化疗药物诱导的凋亡^[9]^。MRP属于三磷酸腺苷依赖性跨膜转运蛋白，其作用机制是通过直接将细胞毒药物排出和/或将药物分离成胞内的小隔离体，使药物不能与靶位点结合，从而导致肿瘤细胞耐药。有报道在小细胞肺癌中，两者的联合表达与耐药密切相关^[3]^。在肺癌细胞中，MRP3 mRNA表达水平与阿霉素、长春新碱、依托泊苷、顺铂耐药密切相关^[10, 11]^。此外，在胰腺癌细胞中MRP3与氟尿嘧啶耐药密切相关；在肝癌细胞中，MRP3与阿霉素耐药密切相关^[12, 13]^。

Anip973/NVB细胞系对多种药物有不同程度的耐药性，细胞耐药性状稳定，是可靠的人肺腺癌MDR细胞模型^[14, 15]^。本实验采用该细胞系建立裸鼠移植瘤模型，成瘤率达到86.7%（13/15）。绘制移植瘤生长曲线发现，Anip973细胞移植瘤经NVB治疗后生长明显减缓甚至缩小，其它三组移植瘤生长无明显差异，生长曲线接近；称瘤质量，计算出Anip973细胞移植瘤经NVB治疗的抑瘤率为60%，生长明显受抑，Anip973/NVB细胞移植瘤的抑瘤率为4.65%，生长没有受到抑制；电镜下，经NVB治疗后Anip973细胞出现凋亡的特征性形态改变，而Anip973/NVB耐药细胞未出现细胞凋亡的表现，NVB对其无抑制作用。以上结果表明Anip973/NVB耐药细胞移植瘤已对NVB耐药。文献^[16]^报道，MRP与多种肿瘤细胞耐药密切相关。亦有研究^[10]^发现MRP的过度表达增强了ATP依赖的GS-X泵的转运活性，从而增加GSH-S偶合物对细胞毒药物的外排，此外MRP还能通过改变胞内药物分布产生耐药；*Bcl-2*是凋亡抑制原癌基因，其编码蛋白具有抑制细胞凋亡作用，并能延长细胞生存期，已证实其在肺癌患者的耐药性中起着重要的作用^[11, 12]^。本实验中，免疫组化结果显示MRP3及Bcl-2在Anip973/NVB耐药细胞移植瘤中的阳性表达率明显高于在Anip973细胞移植瘤中的表达。我们分析可能是由于MRP3通过上述机制致使Anip973/NVB耐药细胞内化疗药物蓄积减少，从而降低了药物对细胞的毒性作用。我们的研究结果提示，*MRP3*基因可能参与了Anip973/NVB耐药细胞系的多药耐药；*Bcl-2*基因可能通过抑制了长春瑞滨诱导的细胞凋亡，从而延长了Anip973/NVB耐药细胞的存活期，这可能也是该细胞系产生耐药的机制之一。此外，经一次NVB治疗后，MRP3及Bcl-2蛋白在Anip973细胞及Anip973/NVB耐药细胞移植瘤中的表达与各自对照组比较无明显差别，提示一次治疗可能没有引起Anip973细胞对NVB耐药，且Anip973/NVB耐药细胞经NVB治疗后没有对MRP3及Bcl-2蛋白的表达产生影响。本研究未发现MRP3及Bcl-2蛋白表达在Anip973/NVB耐药细胞系中的相关性。

多药耐药是一个十分复杂的生物过程，影响因素及参与机制众多，一种化疗药物耐药可能涉及多种耐药基因、蛋白表达的改变。Anip973/NVB细胞系动物模型的建立对今后研究NSCLC对NVB的多药耐药机制具有较大的实用价值，我们以后也可以通过此方法检测其它肿瘤相关因子来研究该细胞系的多药耐药机制。
